# Testing of Anti-EMT, Anti-Inflammatory and Antibacterial Activities of 2′,4′-Dimethoxychalcone

**DOI:** 10.3390/ph17050653

**Published:** 2024-05-17

**Authors:** Peiling Zhao, Mengzhen Xu, Kai Gong, Kaihui Lu, Chen Ruan, Xin Yu, Jiang Zhu, Haixing Guan, Qingjun Zhu

**Affiliations:** 1 Innovative Institute of Chinese Medicine and Pharmacy, Shandong University of Traditional Chinese Medicine, Jinan 250355, China; 17860548018@163.com (P.Z.); 2023100144@sdutcm.edu.cn (M.X.); 2022111508@sdutcm.edu.cn (K.G.); 2022111509@sdutcm.edu.cn (K.L.); 2022110141@sdutcm.edu.cn (X.Y.); 2023111502@sdutcm.edu.cn (J.Z.); 2 School of Pharmacy, Shandong University of traditional Chinese Medicine, Jinan 250355, China; rc18720050881@163.com; 3 Experimental Center, Shandong University of Traditional Chinese Medicine, Jinan 250355, China; 4 Shandong Provincial Key Laboratory of Traditional Chinese Medicine, Shandong University of Traditional Chinese Medicine, Jinan 250355, China; 5 Key Laboratory of Traditional Chinese Medicine Classical Theory, Ministry of Education, Shandong University of Traditional Chinese Medicine, Jinan 250355, China

**Keywords:** chalcone derivatives, 2′,4′-dimethoxychalcone, anti-EMT, anti-inflammatory, antibacterial, pharmacological activity

## Abstract

Chalcone (1,3-diaryl-2-propen-1-one) is an α, β-unsaturated ketone that serves as an active constituent or precursor of numerous natural substances, exhibiting a broad spectrum of pharmacological effects. In this study, the classical Claisen–Schmidt condensation method was used to synthesize the chalcone derivative 2′,4′-dimethoxychalcone (DTC) and evaluate its pharmacological activity. By upregulating the expression of the epithelial cell marker E-cadherin and downregulating the expression of the mesenchymal cell marker vimentin, DTC was found to inhibit transforming growth factor-β1 (TGF-β1)-induced epithelial–mesenchymal transition (EMT) process in A549 cells, maintaining the cells’ epithelial-like morphology and reducing the ability of the cells to migrate. Additionally, DTC demonstrated the ability to decrease the expression levels of nitric oxide (NO), tumor necrosis factor (TNF-α), interleukin-6 (IL-6), and interleukin-1β (IL-1β) in RAW264.7 cells, suggesting a possible anti-inflammatory effect. Furthermore, DTC was found to exhibit bacteriostatic activity against *Staphylococcus aureus* (*S. aureus*), *Proteus vulgaris* (*P. vulgaris*), methicillin-resistant Staphylococcus aureus (MRSA), and *Candida albicans* (*C. albicans*), indicating that this chemical may possess broad-spectrum antibacterial activity.

## 1. Introduction

Chalcone (1,3-diaryl-2-propen-1-one) is the basic skeleton and core of the biological activities of natural products such as isoliquiritigenin, butein, and cardamonin. These natural products have a wide range of pharmacological activities such as antitumor, antifibrotic, anti-inflammatory, antibacterial, etc., and play an important role in the prevention and treatment of many diseases. Epithelial–mesenchymal transition (EMT) plays an important role in tumor metastasis. During the process of EMT, epithelial cells undergo a phenotypic and functional transformation, transitioning into mesenchymal cells and gaining invasive and migratory capabilities [1]. Transforming growth factor-β1 (TGF-β1) is a key factor in the induction of EMT. It has been discovered that isoliquiritigenin reverses the EMT of endometrial carcinogenesis by blocking the TGF-β/Smad signaling pathway, which in turn prevents the migration of endometrial cancer cells [2]. The development of some tumor diseases is closely related to long-term inflammatory stimuli. In the inflammatory response, macrophages secrete large amounts of tumor necrosis factor (TNF-α), interleukin-6 (IL-6), and other pro-inflammatory factors that participate in the body’s immune response. In a mouse model of atherosclerosis, butein reduced the release of pro-inflammatory factors interleukin-1β (IL-1β), IL-6, and TNF-α, thus demonstrating its anti-inflammatory properties [3]. Inflammation is the body’s defensive immune response triggered by bacterial or viral infection, cellular damage, and so on. Isobavachalcone, a natural chalcone derivative, penetrates the outer mold of bacteria and rapidly kills Staphylococcus aureus (MRSA) and *Escherichia coli* [4]. Since chalcone has a straightforward chemical structure, recently, numerous derivatives have been created as lead compounds; two of these are chalcone analogs that have received clinical approval: metochalcone and sofalcone [5].

The substitution of methoxy has attracted much attention in drug discovery and development. Using chalcone as the basic skeleton and substitution of methoxy on the benzene ring attached to the carbonyl group, the derivatives showed higher biological activity, which may be related to the power supply effect of methoxy [6]. Using cinnamaldehyde matrices and groups in the adjacent, interstitial, and para-positions to modify its aromatic ring, Warsito [7] et al. examined the anticancer activity of the derivatives following methoxy and hydroxy substitution. They discovered that methoxy-substituted cinnamaldehyde had higher inhibitory activity against the cancer receptor 4EL9. Fu Peng [8] et al. introduced methoxy on isoliquiritigenin to obtain 3′,4′,5′,4″-tetramethoxychalcone, and the derivatives induced apoptosis through the upregulation of Bax and downregulation of Bcl-2, which in turn inhibited the proliferation of triple-negative breast cancer cells. In the present study, 2′,4′-dimethoxychalcone (DTC) was synthesized by Claisen-Schmidt condensation reaction of 1-(2,4-dimethoxyphenyl) ethanone with benzaldehyde.. Compared with other synthetic methods, this method is simple to operate, has a high rate of carbon–carbon double bond formation, and is easy to purify as the reaction process does not produce a complex mixed structure. When methoxy is attached to the benzene ring, it may transport electrons to the neighboring π-electron system through conjugation, increasing the charge density of the benzene ring structure, improving the bioavailability of DTC, prolonging the duration of action, and enhancing the pharmacological activity. Therefore, we conducted a series of experiments to investigate the pharmacological activity of its derivative, DTC, and preliminarily evaluated the anti-EMT, anti-inflammatory, and antibacterial activities of DTC. This provides a multifaceted reference for the pharmacological study of DTC.

## 2. Results

### 2.1. Chemistry

**Figure 1:**
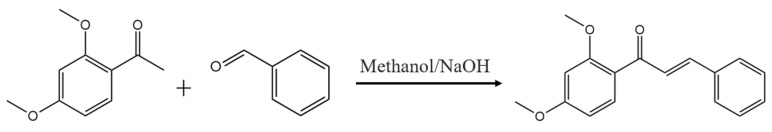
DTC synthetic route.

In this study, DTC was synthesized using the standard Claisen–Schmidt condensation method, which is a reasonably easy way to synthesize an α, β-unsaturated ketone like chalcone [9]. The synthetic route is shown in Figure 1. The structures of the target compounds were confirmed by ^1^H NMR, ^13^C NMR, and HPLC (relevant data are in the Supplementary Materials).

DTC: yellow solid, yield 92.6%. 1H NMR (600 MHz, DMSO-d6) δ 7.77 (d, J = 8.6 Hz, 1H), 7.68 (d, J = 15.8 Hz, 1H), 7.60 (dd, J = 7.6, 1.7 Hz, 2H), 7.52 (d, J = 15.8 Hz, 1H), 7.41–7.36 (m, 3H), 6.57 (dd, J = 8.6, 2.3 Hz, 1H), 6.50 (d, J = 2.3 Hz, 1H), 3.91 (s, 3H), 3.87 (s, 3H). 13C NMR (151 MHz, DMSO-d6) δ 185.77, 159.48, 155.70, 137.26, 128.17, 125.22, 124.10, 123.57, 122.47, 117.49, 100.48, 93.92, 51.03, 50.83 [10].

### 2.2. Assessment of Biology

#### 2.2.1. Effect of DTC on TGF-β1-Induced EMT in A549 Cells

In this part of the experiment, TGF-β1 was employed to stimulate the EMT process in A549 cells, which are human type II alveolar epithelial cells. The anti-EMT effect of DTC was evaluated by experimental methods such as MTT, qRT-PCR, staining of the cell structure, and the cell mobility assay.

The effect of DTC on the viability of A549 cells was first detected by the MTT assay. We set seven sets of concentration gradients for the target compounds at 160, 80, 40, 20, 10, 5 and 0 μM, and five replicates were set for each concentration, and after the cells were treated with different working concentrations for 48 h, the IC_10_ value was calculated by using an enzyme marker to read the absorption value at 490 nm. The findings are displayed in Table 1, and the IC_10_ of DTC was 23.89 μM.

A decrease in epithelial cells and an increase in mesenchymal cells characterize the EMT process. In this study, real-time fluorescence quantitative PCR was used to detect the effect of DTC on the TGF-β1-induced mRNA expression of epithelial cell marker E-cadherin and mesenchymal cell marker vimentin genes of A549 cells and to assess the DTC inhibitory effect on the EMT process.

**Figure 2:**
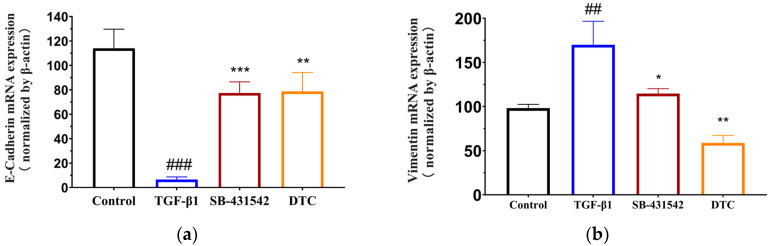
DTC’s effect on vimentin and E-cadherin mRNA expression in A549 cells following TGF-β1 stimulation. Blank control group: control group; Model group: TGF-β1 group; Positive control group: SB-431542; Experimental group: DTC group. After TGF-β1 (5 ng/mL) treatment, A549 cells were treated with DTC (23.89 μM) and SB-431542 (10 μM) for 48 h. The cells were then assayed using qRT-PCR to detect the expression of E-cadherin mRNA (**a**) versus vimentin mRNA (**b**), and the data were analyzed for relative quantification (means ± SEM, *n* = 3). ^###^ *p* < 0.001, ^##^ *p* < 0.01 vs. control group; *** *p* < 0.001, ** *p < 0*.01, * *p <* 0.05 vs. TGF-β1 model group.

Figure 2 displays the findings of the qRT-PCR analysis. In comparison to the blank control group, the cells in the model group underwent EMT after TGF-β1 induction, with a decrease in the expression of mRNA for the epithelial cell marker E-cadherin gene and an increase in the expression of mRNA for the mesenchymal cell marker vimentin gene. Following the intervention of SB-431542 and DTC, compared with the model group, the relative expression of the E-cadherin gene mRNA in A549 cells was significantly higher, while the relative expression of the vimentin gene mRNA was significantly reduced. As a result, DTC can inhibit TGF-β1-induced EMT in A549 cells.

A549 cells underwent a transformation from cobblestone-like to long spindle-shaped with an increased cell length and area during TGF-β1-induced EMT. We dyed the cells to examine the impact of DTC on the morphology and structure of A549 cells caused by TGF-β1.

**Figure 3:**
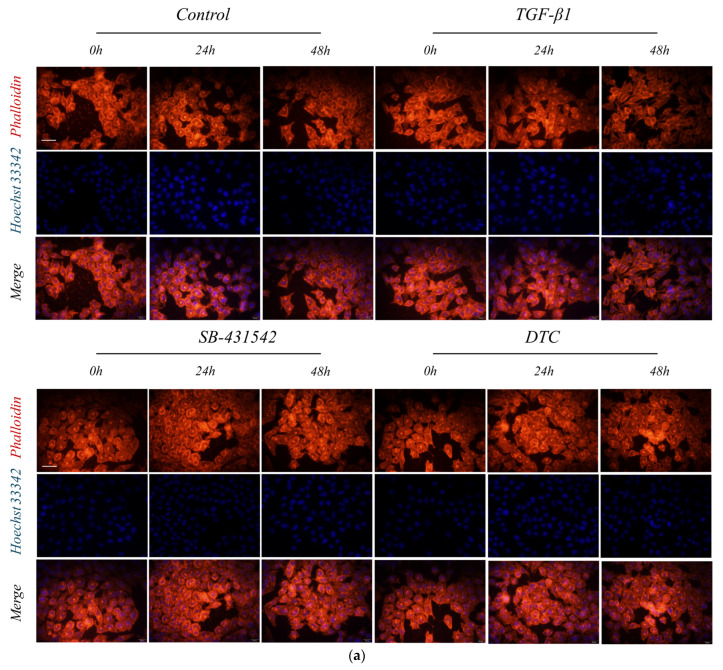

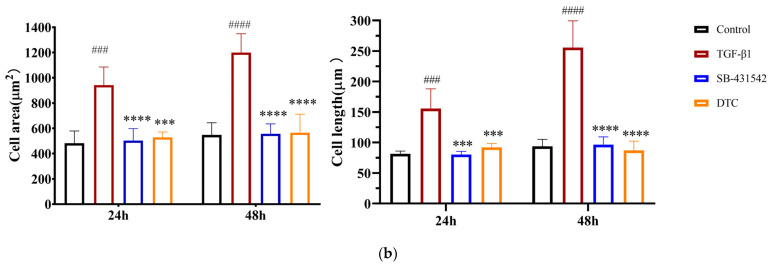
DTC’s effect on the morphology of A549 cells following TGF-β1 stimulation. Blank control group: control group; Model group: TGF-β1 group; Positive control group: SB-432542; Experimental group: DTC group. TGF-β1 (5 ng/mL), DTC (23.89 μM), and SB-431542 (10 μM) were added to A549 cells for 24 and 48 h of treatment. TRITC-phalloidin (red) was used to label the cytoplasm, while Hoechst 33342 (blue) was used to identify the nucleus. Fluorescence microscopy was used to photograph the cells (**a**) with a scale bar of 20 μm. ImageJ 1.52a was used to estimate the cytosolic length and area (**b**), and the data were quantitatively analyzed (means ± SEM, *n* = 6). ^####^ *p* < 0.0001, ^###^ *p* < 0.001 vs. control group; **** *p* < 0.0001, *** *p* < 0.001 vs. TGF-β1 model group.

Representative images of the cell morphology and structure staining and analysis results are shown in Figure 3. The cell morphology of the blank control group was irregular polygonal with cobblestone morphology. After TGF-β1 stimulation, we observed that the morphology of A549 cells was long and spindle-shaped, with a notable increase in cell area and length, exhibiting the morphological features of mesenchymal cells in comparison to the blank control group. After 48 h of intervention with SB-431542 and DTC, the cell length and area were reduced to different degrees compared with the model group, showing the typical cobblestone-like morphology of epithelial cells. The findings demonstrate that DTC might prevent A549 cells from changing morphologically following TGF-β1 stimulation.

**Figure 4:**
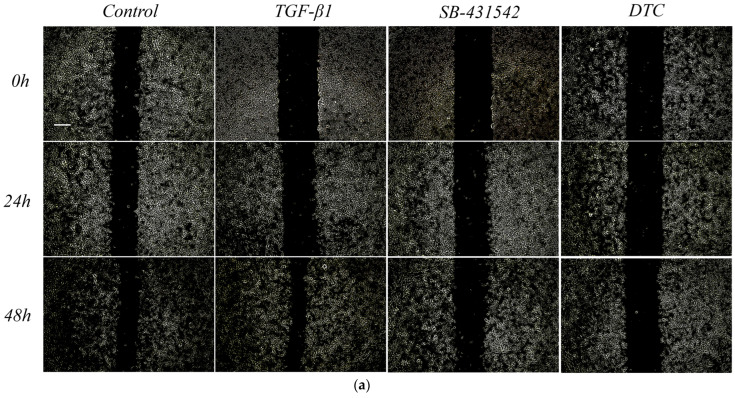

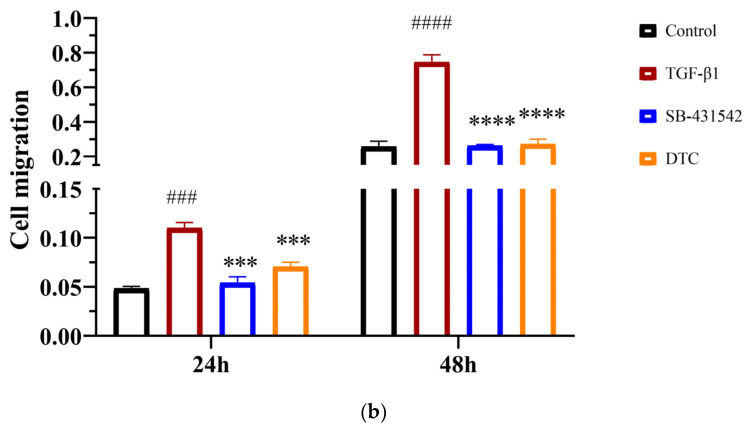
Impact of DTC on the ability of the A549 cells to migrate following TGF-β1 stimulation. Blank control group: control group; Model group: TGF-β1 group; Positive control group: SB-432542; Experimental group: DTC group. TGF-β1 (5 ng/mL), DTC (23.89 μM), and SB-431542 (10 μM) were added to A549 cells for 24 and 48 h of treatment. Acquisition of images with an inverted phase contrast microscope and color conversion of photographs using ImageJ (**a**), scale bar: 2 mm; the differential change in scratch area was measured (**b**) and quantitatively analyzed by applying ImageJ software (means ± SEM, *n* = 3). ^####^ *p* < 0.0001, ^###^ *p* < 0.001 vs. control group; **** *p* < 0.0001, *** *p* < 0.001 vs. TGF-β1 model group.

During TGF-β1-induced EMT, A549 cells lost apical-basal polarity and cell migration was enhanced. Based on the obvious effect of DTC in inhibiting TGF-β1-induced EMT of A549, we further investigated their effects on the migration ability of TGF-β1-induced A549 cells. The results are shown in Figure 4, where compared with the blank control group, the area of the scratched area was significantly reduced and the migration ability of the cells was enhanced after 24 h and 48 h of TGF-β1-induced A549 cells. Compared with the model group, SB-431542 and DTC were able to attenuate the TGF-β1-induced migration ability of the A549 cells.

#### 2.2.2. Effect of DTC on Inflammatory Factors in LPS-Stimulated RAW264.7 Cells

The mouse macrophage RAW264.7 was chosen as the study subject for this experiment, and the impact of DTC on the viability of RAW264.7 cells was determined using MTT. Inflammatory responses were induced in RAW264.7 cells using lipopolysaccharide (LPS), NO levels in the cell supernatants were quantified by the Griess method [11], and TNF-α, IL-1β, and IL-6 levels in the cell supernatants were measured by ELISA.

First, the MTT method was used to evaluate the impact of DTC on the viability of RAW264.7 cells. The working concentrations of the compounds were set at 80, 40, 20, 5, 2.5, and 0 μM with five replicate experiments for each set of concentrations. The cells were treated with the drug concentration for 48 h, and finally, the data of the absorption values were obtained with the help of an enzyme marker at a 490 nm wavelength and further analyzed based on the formula to calculate the cell viability.

**Figure 5:**
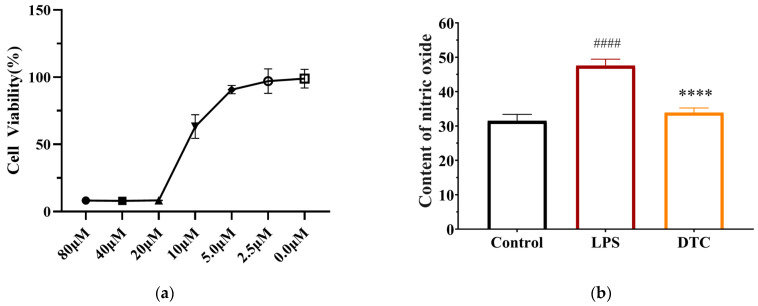
(**a**) Effect of DTC on RAW264.7 cell viability. After 48 h of treatment with 80 μM, 40 μM, 20 μM, 10 μM, 5 μM, 2.5 μM, and 0 μM, cell viability was assessed by MTT. (**b**) Effect of DTC on NO production in the supernatant of RAW264.7 cells. RAW264.7 cells were divided into the blank control group (without LPS), LPS group (cells treated with 1 μg/mL LPS for 48 h), and DTC group (1 μg/mL + 5 μM DTC treatment for 48 h), and the NO content in the cell supernatant was determined by the Griess method. ^####^ *p* < 0.0001 vs. control group; **** *p* < 0.0001 vs. TGF-β1 model group.

We chose 5.0 μM as the DTC working concentration because, as Figure 5a demonstrates, there was no cytotoxicity to the RAW264.7 cells when the working concentration was below 5.0 μM. Figure 5b displays the effects of DTC on NO in the supernatant following LPS induction. The LPS model group’s cell supernatant NO content increased significantly in comparison to the blank control group, while the DTC group’s cell supernatant NO content decreased following the intervention.

**Figure 6:**
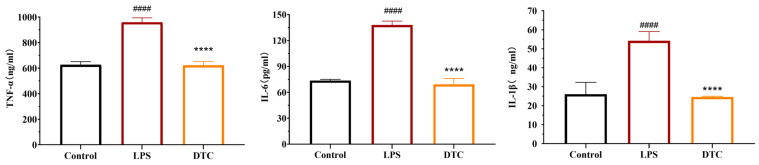
Effect of DTC on the LPS-induced TNF-α, IL-6, and IL-1β levels in the supernatant of RAW264.7 cells. RAW264.7 cells were divided into the blank control group (without LPS), LPS group (cells treated with 1 μg/mL LPS for 48 h), and DTC group (1 μg/mL + 5 μM DTC treatment for 48 h), and determination of TNF-α, IL-6 and IL-1β levels in the cell supernatants. ^####^ *p* < 0.0001 vs. control group; **** *p* < 0.0001 vs. TGF-β1 model group.

We also evaluated the impact of DTC on the TNF-α, IL-6, and IL-1β levels in the RAW264.7 cell supernatant. Figure 6 illustrates the considerable rise in TNF-α, IL-6, and IL-1β expression levels in the LPS model group compared to the control group. After the DTC intervention, there was a significant decrease in the expression levels of TNF-α, IL-6, and IL-1β in the cell supernatant compared to the LPS group. It was shown that DTC was able to reduce the expression levels of TNF-α, IL-6 and IL-1β in the supernatant of the LPS-induced RAW264.7 cells.

#### 2.2.3. Bacteriostatic Activity of DTC

In this experiment, DTC was determined against *S. aureus*, *Pseudomonas aeruginosa* (*P. aeruginosa*), *P. vulgaris*, *Escherichia coli* (*E. coli*) using micro broth dilution method, MRSA, *C. albicans*, *Gardnerella vaginalis* (*G. vaginalis*), *Streptococcus agalactiae* (*S. agalactiae*), *Enterococcus faecalis* (*E. faecalis*), *Bacillus subtilis* (*B. subtilis*) with the minimum inhibitory concentration (MIC) and minimum bactericidal concentration (MBC). The test results are shown in Table 2, where DTC showed bacteriostatic activity against *S. aureus*, *P. vulgaris*, MRSA, and *C. albicans*, in which the bacteriostatic activity against *C. albicans* was significantly stronger than that of the other bacteria, with an MIC of 10 μM and MBC of 12.5 μM.

## 3. Discussion

Tumor migration and invasion, tissue repair, wound healing, and embryonic development are all impacted by the crucial cell biology process known as EMT [12]. Epithelial cells undergo a loss of their phenotypic characteristics and functionality during EMT, transitioning into mesenchymal cells and gaining the ability to invade and migrate [1]. It has been demonstrated that TGF-β1 is a key factor in the induction of EMT, which promotes the apoptosis and migration of epithelial cells, activation of fibroblasts, proliferation and transformation of myofibroblasts, and induces epithelial EMT via the TGF-β/Smads pathway [13]. There are three main types of this biological behavior. Type I is involved in the implantation and growth of embryos; type II promotes wound healing; and in type III, a few cancer cells in the primary tumor cells trigger EMT, changing their epithelial characteristics into mesenchymal characteristics, decreasing intercellular adhesion, and increasing invasiveness to promote the spread of cancer cells from the original site to other sites. In addition, EMT has the property of inducing stem cells, which enhances the self-renewal ability and differentiation potential of tumor cells and makes tumor cells resistant to chemotherapy, radiotherapy, and immunotherapy, etc., in order to facilitate the survival and metastasis of tumor cells in the host body [14,15,16]. In this study, we used TGF-β1 to induce EMT in type II alveolar epithelial cells A549, and the experimental results showed that when the working concentration of DTC was 23.89 μM, it was able to significantly increase the expression of epithelial cell marker E-cadherin, decrease the expression of mesenchymal cell marker vimentin, diminish the migration ability of the cells, and maintain the epithelial-like morphology of the cells. It also significantly inhibited TGF-β1-induced EMT in the A549 cells.

It is commonly known that a protracted inflammatory response is intrinsically linked to the development of certain oncological illnesses, and that macrophages play a critical role in both starting and participating in this response. LPS is an important component of the cell wall of Gram-negative bacteria, which can cause inflammation, shock, and infection in the body, and its induction of RAW264.7 macrophages to produce an inflammatory response is a classical inflammatory cell model [17]. In order to evaluate the anti-inflammatory activity of DTC, an in vitro inflammation model was constructed to measure the NO, IL-6, IL-1β, and TNF-α levels in the cell supernatants. It was found that DTC at a working concentration of 5 μM was able to inhibit the LPS-induced secretion of NO, IL-6, IL-1β, and TNF-α in the supernatant of the RAW264.7 cells, exerting anti-inflammatory effects. Some studies have shown that chalcone derivatives have bacteriostatic activity such as inhibitory effects of ferrocenyl chalcone derivatives and dihydrochalcone compounds on Gram-positive bacteria MRSA [18,19] as well as the inhibition of Pseudomonas aeruginosa by (2E)-1-(5-methylfuran-2-yl)-3-(4-nitrophenyl) prop-2-en-1-one [20]. However, the bacterial species in these studies were homogeneous. Our study revealed that DTC inhibited *S. aureus*, *P. mirabilis*, MRSA and *C. albicans*, suggesting that DTC has excellent broad-spectrum antimicrobial activity.

In summary, our study found that DTC exhibited low toxicity, high bioavailability, and excellent pharmacological activity in the in vitro experiments. This shows the broad application prospect of DTC and provides a basis for the development of related drugs.

## 4. Materials and Methods

### 4.1. Chemistry

A 250 mL round-bottomed flask containing 60 mL of methanol was filled with benzaldehyde (6.00 mmol) and allowed to dissolve under magnetic stirring. Next, 480 mg of sodium hydroxide (12 mmol) was added gradually, and after the mixture was dissolved, 6.15 mmol of 2′,4′-dimethoxyacetophenone was added. The reaction was then run for six hours at reflux. In alkaline environments, DTC is generated by dehydration of hydroxyl aldehyde products via an enol salt mechanism. Due to the conjugated double-bond structure of chalcone, DTC has fluorescent properties, and thus the reaction process can be detected by the thin-layer chromatography (TLC) of TCL silica gel 60 F254 (0.20 mm; Qingdao Oceanic Chemical Factory, Qingdao, China) together with its fluorescent properties. After the reaction was completed, the pH was adjusted to 1.0–2.0 with dilute hydrochloric acid, and the product was extracted with ethyl acetate and finally separated by silica gel column chromatography (the mobile phase was ethyl acetate-petroleum ether). The synthesized DTC was used as a solvent with DMS0-d6 to record the ^1^H and ^13^C nuclear magnetic resonance (NMR) spectra on a Bruker Avance 400 spectrometer (Bruker, Germany). HPLC was conducted to verify the purity of the compounds.

### 4.2. Biological Assays for Anti-EMT

#### 4.2.1. Cell Culture and Drug Configuration

A549 cells (MeilunBio, Dalian, China) were grown in RPMI-1640 (Gibco, Sigma Aldrich, Søborg, Denmark), which included 1% penicillin-streptomycin and 10% fetal bovine serum (Gibco, Sigma Aldrich, Søborg, Denmark). Kept in an incubator with 5% CO_2_ at 37 °C, the chemical was combined with dimethyl sulfoxide (DMSO) to create a 20 mmol/L solution, which was then kept at −20 °C.

#### 4.2.2. MTT Assay

We adjusted the cell density to 5 × 10^4^ cells/mL per well and added 150 μL per well to a 96-well plate (PerkinElmer, Waltham, MA, USA) and then placed them in an incubator for 24 h. We set seven groups of concentration gradients for the target compound, which were 160, 80, 40, 20, 10, 5, and 0 μM, and set five replicates for each concentration. After 48 h of cell treatment at different working concentrations, 90 uL of culture medium and 10 uL of MTT solution (Solabio, Beijing, China) were added to each well and incubated for 4 h in the dark. After aspirating the supernatant, 110 microliters of dimethyl sulfoxide solution were added to each well. The solution was dissolved by shaking on a shaker at low speed for 10 min, and the absorbance (OD) of each hole was measured at 490 nm. The absorbance (OD) of each well was measured at 490 nm, and the cell viability was calculated according to the formula.

#### 4.2.3. qRT-PCR Assay

To measure the impact of target drugs on the TGF-β1-induced mRNA expression of genes associated with EMT in A549 cells, the cells were separated into four groups: DTC, TGF-β1 group, blank control group, and SB-431542. Using an RNA extraction kit (Vazyme, Nanjing, China), the cell RNA was extracted following a 48-h intervention. HiScript III RT Super Mix for qPCR (Vazyme, Nanjing, China) was used in a two-step procedure to create first-strand DNA, and the Taq Pro Universal SYBR qPCR Master Mix kit was used to amp up qPCR. Fluorescence quantitative PCR used a 20 μL reaction system, and 2^−ΔΔCt^ was used to quantify the relative expression levels of vimentin and E-cadherin mRNA. The primer sequences are listed in Table 3.

#### 4.2.4. Cell Staining

A549 cells were inoculated into a 96-well plate at the rate of 100 μL per well (containing 5 × 10^3^ cells), and the peripheral holes were filled with phosphate buffer solution. After 24 h of culturing, A549 cells were treated with TGF-β1 (5 ng/mL) and DTC (23.89 μM). The experimental cells exposed to drugs for 0 h, 24 h, and 48 h were stained, respectively. The cell culture medium in the 96-well plate was sucked out, washed twice with PBS, and 100 μL 4% paraformaldehyde (Solabio, Beijing, China) was put into each well to fix the cells at room temperature for 15 min. The stationary solution was sucked out before it was rinsed with PBS 3 times, then we put 100 μL of 0.1% Tritonx-100 (Sinopharm, Shanghai, China) into each hole, let it stand at room temperature for 10 min to increase the permeability, and rinsed it again with PBS 3 times. We then added 150 μL TRITC-phalloidin (Solabio, Beijing, China) working solution to each well, incubated it at room temperature for 30 min, and rinsed with PBS twice. Each well was stained with 150 μL Hoechest 33342 (Solabio, Beijing, China) for 5 min, washed with PBS twice, and observed under a fluorescence microscope.

#### 4.2.5. Assay for Cell Scratches

We adjusted the density of the A549 cell suspension to 3 × 10^5^/mL, added 2 mL per well into a 6-well plate, and then placed it in an incubator. After 24 h of cell culture, a 200 μL micro-sampler head was used to scratch the bottom wall of the orifice plate, keeping the same strength and angle to ensure the same scratch width between different holes, and then PBS was used to clean it three times after scratching. A549 cells, treated with TGF-β1 (5 ng/mL) and DTC (23.89 μM), were intervened for 24 h and 48 h, respectively, with 2 mL per hole. These were then observed under a microscope and the scratch areas recorded at 0 h, 24 h and 48 h.

𝑀 = (𝑆𝑎 − 𝑆𝑒)/𝑆𝑎(1)

*M* = migration ability, *Sa* = cell scratch area of 0 h, *Se* = cell scratch area of 24/48 h.

### 4.3. Biological Assays for Anti-Inflammation

#### 4.3.1. Cell Culture and Drug Configuration

RAW264.7 cells (MeilunBio, Dalian, China) were grown in DMEM (Gibco, Sigma Aldrich, Søborg, Denmark), which included 1% penicillin-streptomycin and 10% fetal bovine serum (Gibco, Sigma Aldrich, Søborg, Denmark). These were kept in an incubator with 5% CO_2_ at 37 °C, then the chemical was combined with dimethyl sulfoxide (DMSO) to create a 20 mmol/L solution, which was then kept at −20 °C.

#### 4.3.2. MTT Assay

The effect of DTC on the activity of the RAW264.7 cells was determined by the MTT method. After adjusting the cell density to 6 × 10^3^ cells/mL per well and adding 150 μL per well to a 96-well plate, we placed them in the incubator for 24 h. We set seven groups of concentration gradients for the target compound, which were 80, 40, 20, 10, 5, 2.5 and 0 μM, and set five replicates for each concentration. After 48 h of cell treatment at different working concentrations, 90 uL of the culture medium and 10 uL of the MTT solution were added to each well and incubated for 4 h in the dark. After aspirating the supernatant, 110 microliters of dimethyl sulfoxide solution was added to each well. The solution was dissolved by shaking on a shaker at low speed for 10 min, and the absorbance (OD) of each hole was measured at 490 nm. The absorbance (OD) of each well was measured at 490 nm, and the cell viability was calculated according to the formula.

#### 4.3.3. Effect of DTC on NO Content in RAW264.7 Cell Supernatant by Griess Method

The cell density was adjusted to 1 × 10^6^ cells/mL, and 100 µL per well was added to a 96-well plate (PerkinElmer, Waltham, MA, USA) and placed in an incubator for 24 h. The RAW264.7 cells were split into three groups: a blank control, a model group (1 μg/mL LPS), and an experimental group (5 μM DTC +1 μg/mL LPS). The cells were then incubated for 48 h at a constant temperature of 37 °C with 5% CO_2_. The cell supernatants were quantified using the Griess method. A total of 50 μL of the cell supernatant was aspirated from each well in a new 96-well plate, and then 50 μL of Griess A and Griess B were added, respectively, and incubated at room temperature for 10 min. Absorbance was detected at a light wavelength of 540 nm, and the concentration of NO was calculated in each sample according to the standard curve of NaNO_2_ dilution.

#### 4.3.4. Detection of TNF-α, IL-1β and IL-6 Levels in Cell Supernatants Using ELISA Kits

The density of the RAW264.7 cells was made into 1 × 10^5^ cells/mL, inoculated in 96-well plates, incubated for 24 h to make them adherent to the wall, then grouped according to the settings of 4.3.3. Next, the cell supernatant was collected after 48 h of incubation and then centrifuged at 3000 r/min for 10 min. The supernatant was aspirated, and the absorbance was measured at the wavelength of 450 nm.

### 4.4. Assays for Antimicrobial Biologicals

#### 4.4.1. Bacterial Activation and Culture

The strains were removed from a −80 °C refrigerator, placed in a 37 °C-water bath to melt, and the appropriate amount of bacterial solution was coated in a solid Petri dish, placed in a 37 °C bacterial incubator for 24 h, and then stored at 4 °C for spare use. The activated bacteria were inoculated in the liquid medium and placed in a bacterial incubator at 37 °C for 24 h. The bacterial solution was diluted with liquid medium to 10^7^–10^8^ CFU/mL before the experiment. The source of the strains is presented in Table 4, and the names of the culture media and their manufacturers are in Table 5.

#### 4.4.2. MIC and MBC of DTC

A total of 100 μL of DTC-containing liquid medium at different concentrations was added to 96-well plates, and a negative control group without drugs and a positive control group with antibiotics were set up. A total of 20 μL of bacterial solution was added to all groups, mixed, and incubated at 37 °C on a shaker for 24 h. Then, 20 μL of LTTC was added to each group and incubated for 2 h protected from light, and the minimum dilution concentration for the sterile growth test was measured as the MIC by measuring the OD value of the enzyme marker. The aseptically grown target compound bacterial solution was coated and inoculated in solid medium and incubated at 37 °C for 24 h. The minimum concentration of aseptic growth within the solid medium plate was taken as the MBC.

### 4.5. Analysis of Statistics

The experimental data in this study were statistically analyzed and graphed using GraphPad Prism 9.0 software. The quantitative data were shown as the mean ($\bar{x}\pm s$) and standard deviation. To analyze the various sample groups, one-way analysis of variance (ANOVA) was used, and for pairwise comparisons, it was supplemented with the LSD method. Differences were considered statistically significant at *p* < 0.05. Every test was carried out three times to ensure the reliability of the experimental data.

## 5. Conclusions

In conclusion, chalcone and its derivatives have a wide range of applications, but most of the studies have only evaluated them for a single activity. When methoxy is attached to the benzene ring, it may transport electrons to the neighboring π-electron system through conjugation, increasing the charge density of the benzene ring structure, which in turn increases the bioavailability and pharmacological activity of DTC. Based on this, we evaluated the anti-EMT, anti-inflammatory, and antimicrobial activities of the methoxy-substituted chalcone derivative DTC. The results showed that DTC inhibited the cellular EMT process by upregulating the epithelial cell marker E-cadherin and decreasing the expression of the mesenchymal cell marker vimentin, maintaining a cellular epithelial-like morphology, and decreasing the cellular migration ability. Furthermore, DTC exhibited anti-inflammatory effects by reducing the expression levels of NO, TNF-α, IL-6, and IL-1β in the RAW264.7 cells after LPS induction. In addition, we found that DTC has broad-spectrum antimicrobial effects as well as bacteriostatic activity against *S. aureus*, *P. vulgaris*, MRSA, and *C. albicans*. This study investigated the wide range of pharmacological properties of DTC through comprehensive multi-indicator testing, providing a basis for the development and utilization of DTC.

## Figures and Tables

**Table 1:** IC_10_ value for DTC.

Compound	Structure	Antagonist IC_10_ (μM)
DTC	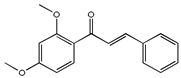	23.89

**Table 2:** MIC and MBC of DTC.

Strains	MIC (μM)	MBC (μM)
*S. aureus*	12.5	15
*P. aeruginosa*	--	--
*P. vulgaris*	15	20
*E. coli*	--	--
MRSA	15	20
*C. albicans*	10	12.5
*G. vaginalis*	--	--
*S. agalactiae*	--	--
*E. faecalis*	--	--
*B. subtilis*	--	--

**Table 3:** Primer sequences for use in qRT-PCR.

Gene Name		Primer Sequence (from 5′ to 3′)
E-Cadherin	Forward	5-GAGTGCCAACTGGACCATTCAGTA-3′
	Reverse	5′-CACAGTCACACACGCTGACCTCTA-3′
Vimentin	Forward	5′-TGACATTGAGATTGCCACCTACAG-3′
	Reverse	5′-TCAACCGTCTTAATCAGAAGTGTCC-3′
β-actin	Forward	5′-TGACGTGGACATCCGCAAAG-3′
	Reverse	5′-CTGGAAGGTGGACAGCGAGG-3′

**Table 4:** The source of the strains.

Name	Source
*S. aureus* (ATCC: 23235)	Courtesy of Department of Microbiology, Shandong University of Traditional Chinese Medicine
*P. aeruginosa* (ATCC: 27853)	Courtesy of Department of Microbiology, Shandong University of Traditional Chinese Medicine
*P. vulgaris* (ATCC: 49132)	Courtesy of Department of Microbiology, Shandong University of Traditional Chinese Medicine
*E. coli* (ATCC: 25922)	Courtesy of Department of Microbiology, Shandong University of Traditional Chinese Medicine
MRSA (ATCC: BAA-172)	Courtesy of Department of Microbiology, Shandong University of Traditional Chinese Medicine
*C. albicans* (ATCC: 18804)	Courtesy of Department of Microbiology, Shandong University of Traditional Chinese Medicine
*G. vaginalis* (ATCC: 49145)	Courtesy of Department of Microbiology, Shandong University of Traditional Chinese Medicine
*S. agalactiae* (ATCC: 13813)	Courtesy of Department of Microbiology, Shandong University of Traditional Chinese Medicine
*E. faecalis* (ATCC: 700802)	Courtesy of Department of Microbiology, Shandong University of Traditional Chinese Medicine
*B. subtilis* (ATCC: 6051)	Courtesy of Department of Microbiology, Shandong University of Traditional Chinese Medicine

**Table 5:** The culture media and their manufacturers.

Name	Source
Sabouraud Dextrose Broth Medium	Qingdao Hi-Tech Industrial Park Haibo Biotechnology Co. (Qingdao, China)
Columbia Broth	Shandong Top Biological Engineering Co. (Yantai, China)
MH	Qingdao Hi-Tech Industrial Park Haibo Biotechnology Co.

## Data Availability

Data will be provided upon request.

## Supplementary Materials

The following supporting information can be downloaded at: https://www.mdpi.com/article/10.3390/ph17050653/s1. Figure S1. ^1^H NMR spectra of compound **2**′**,4**′**-dimethoxychalcone**; Figure S2. ^13^C NMR spectra of compound **2**′**,4**′**-dimethoxychalcone**; Figure S3. HPLC of **2**′**,4**′**-dimethoxychalcone**; Figure S4. Effect of **2**′**,4**′**-dimethoxychalcone** 48h on the activity of A549 cells.
