# Supplementary material for: Testing of Anti-EMT, Anti-Inflammatory and Antibacterial Activities of 2′,4′-Dimethoxychalcone
Source: Pharmaceuticals (Basel). 2024 May 17;17(5):653. doi: 10.3390/ph17050653 (PMC11124480; doi:10.3390/ph17050653)
Supplement: Supplementary file 1 [file pharmaceuticals-17-00653-s001.zip › pharmaceuticals-2990742-supplementary.pdf]

Supplementary Material

Peiling Zhao [1,†], Mengzhen Xu [1,†], Kai Gong[1], Kaihui Lu[1], Chen Ruan[2], Xin Yu[1], Jiang Zhu[1], Haixing Guan [3,4,5,*] and Qingjun Zhu [1,5,*]

1 Innovative Institute of Chinese Medicine and Pharmacy, Shandong University of Traditional Chinese Medicine, Jinan 250355, China;

2 School of Pharmacy, Shandong University of traditional Chinese Medicine, Jinan 250355, China;

3 Experimental Center, Shandong University of Traditional Chinese Medicine, Jinan 250355, China;

4 Shandong Provincial Key Laboratory of Traditional Chinese Medicine, Shandong University of Traditional Chinese Medicine, Jinan 250355, China;

5 Key Laboratory of Traditional Chinese Medicine Classical Theory, Ministry of Education, Shandong University of Traditional Chinese Medicine, Jinan 250355, China；

† These authors contributed equally to this work.

* Correspondence: haixingguan@163.com(H.G.) , zhuqingjunhotmail@gmail.com(Q.Z.).

Table and Figure Caption

Figure S1.^{1}H NMR spectra of compound **2',4'-dimethoxychalcone**

Figure S2. ^{13}C NMR spectra of compound **2',4'-dimethoxychalcone**

Figure S3. HPLC of **2',4'-dimethoxychalcone.**

Figure S4. Effect of **2',4'-dimethoxychalcone** 48h on the activity of A549 cells

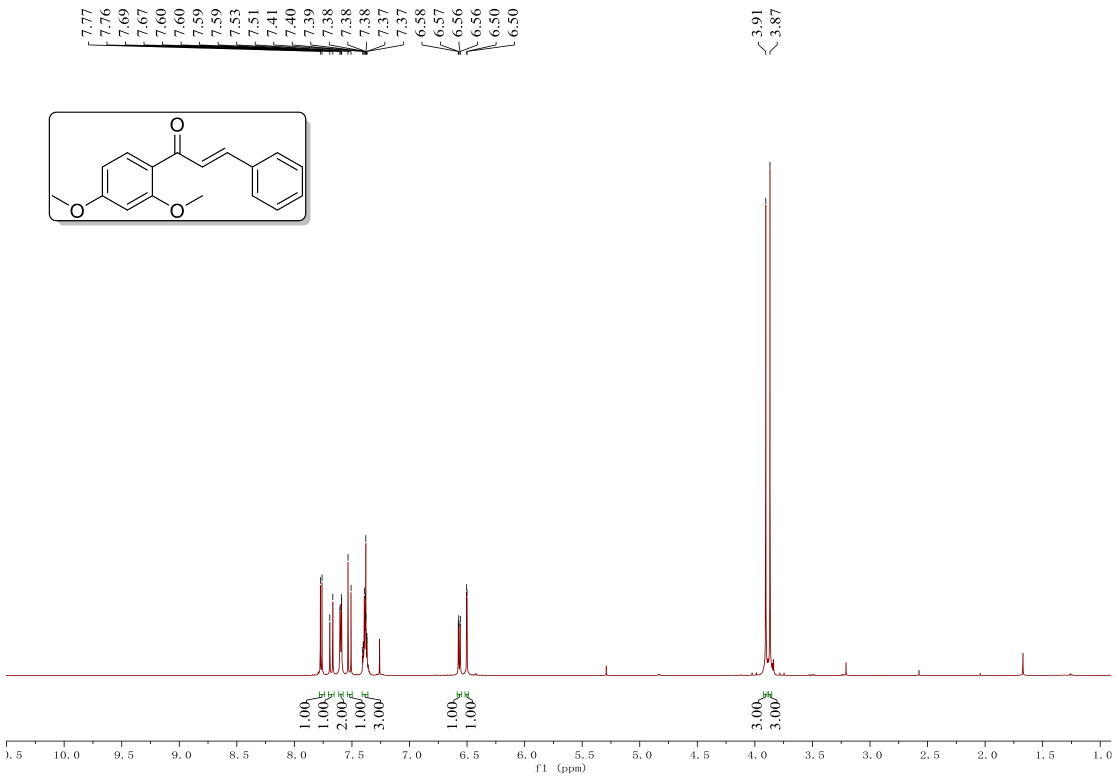

Figure S1.¹H NMR spectra of **2',4'-dimethoxychalcone**

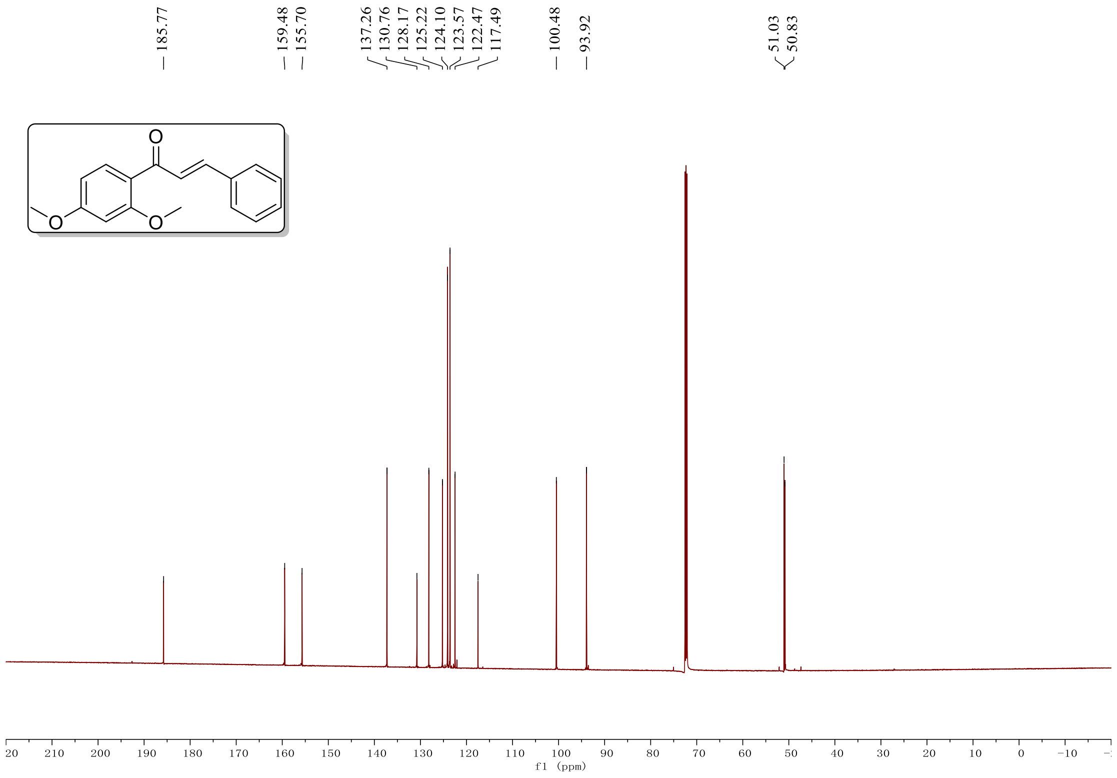

Figure S2. ¹³C NMR spectra of **2',4'-dimethoxychalcone**

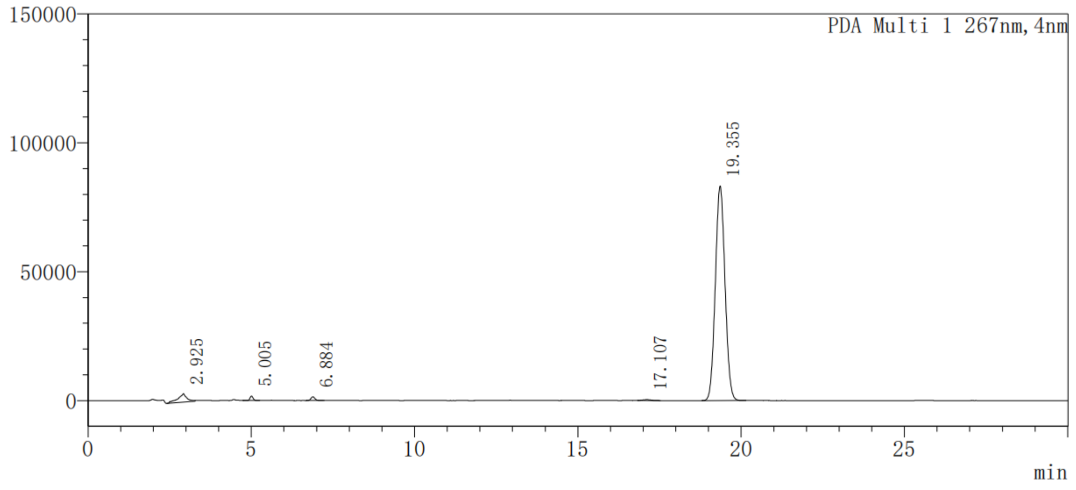

Figure S3. HPLC of **2',4'-dimethoxychalcone.**

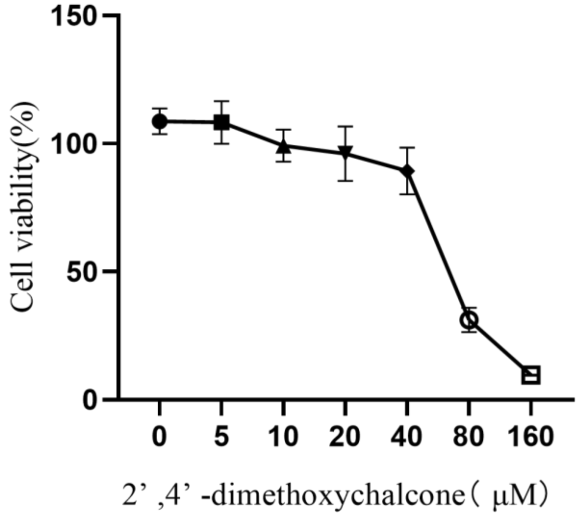

Figure S4. Effect of **2',4'-dimethoxychalcone** 48h on the activity of A549 cells
